# Mediators of Inflammation in Acute Kidney Injury

**DOI:** 10.1155/2009/137072

**Published:** 2010-02-21

**Authors:** Ali Akcay, Quocan Nguyen, Charles L. Edelstein

**Affiliations:** Division of Renal Diseases and Hypertension, University of Colorado Denver, Aurora, CO 80262, USA

## Abstract

Acute kidney injury (AKI) remains to be an independent risk factor for mortality and morbidity. Inflammation is now believed to play a major role in the pathopathophysiology of AKI. It is hypothesized that in ischemia, sepsis and nephrotoxic models that the initial insult results in morphological and/or functional changes in vascular endothelial cells and/or in tubular epithelium. Then, leukocytes including neutrophils, macrophages, natural killer cells, and lymphocytes infiltrate into the injured kidneys. The injury induces the generation of inflammatory mediators like cytokines and chemokines by tubular and endothelial cells which contribute to the recruiting of leukocytes into the kidneys. Thus, inflammation has an important role in the initiation and extension phases of AKI. This review will focus on the mediators of inflammation contributing to the pathogenesis of AKI.

## 1. Introduction

Acute kidney injury (AKI) occurs in 1% of hospital admissions [1] and up to 7% of hospitalized patients develop AKI [1]. Twenty five percent of patients in the intensive care unit (ICU) develop AKI and 5% of patients in the ICU will need renal replacement therapy [1, 2]. AKI is usually diagnosed by increases in serum creatinine or blood urea nitrogen. Recently urinary biomarkers like interleukin-18 (IL-18), kidney injury molecule-1 (KIM-1), and neutrophil gelatinase-associated lipocalin (NGAL) have been used for the early diagnosis of AKI [3]. Dialysis is the only Federal Drug Administration- (FDA-) approved treatment for AKI [4]. Even though both intermittent hemodialysis (IHD) and continuous renal replacement therapy (CRRT) are widely used, the mortality of AKI is as high as 80% in ICU patients [5, 6]. A better understanding of the inflammatory response in AKI is needed to allow interventions which would prevent the need for hemodialysis, shorten the course of AKI, reduce distant organ injury in AKI, and improve survival. 

**Figure 1:**
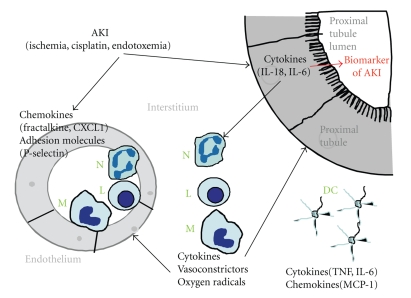
*Inflammatory pathogenesis of AKI*. AKI results in the upregulation of chemokines like fractalkine and CXCL1 and adhesion molecules like P-selectin in the endothelium of blood vessels in the kidney. Upregulation of chemokines and adhesion molecules in the endothelium results in the infiltration of inflammatory cells like neutrophils (N), lymphocytes (L), and macrophages (M) from the blood vessels into the interstitium of the kidney. Proximal tubular epithelial cells produce cytokines like IL-18 and IL-6. IL-18 and IL-6 produced by the proximal tubule enter the interstitium and result in activation and/or proliferation of neutrophils (N), lymphocytes (L), and macrophages (M). IL-18 and IL-6 are also released from the proximal tubular cells into the tubular lumen where they serve as early urinary diagnostic biomarkers of AKI. Inflammatory cells produce vasoconstrictors (prostaglandins, leukotrienes, and thromboxanes) that may affect vascular injury and oxygen radicals that may worsen tubular and vascular injury. Resident dendritic cells (DCs) form a contiguous network throughout the entire kidney. The role of DCs in AKI is not well understood. Following kidney ischemia-perfusion, resident dentritic cells release cytokines like TNF and IL-6 and chemokines like MCP-1.

AKI remains to be an independent risk factor for mortality and morbidity. AKI increases the risk of death in patients after thoracoabdominal aortic surgery [7], after bone marrow transplantation [8], after amphotericin B therapy [9], following cardiac surgery [10], and in patients with liver cirrhosis [11]. An increase in serum creatinine is a risk factor for mortality in hospitalized [12] and ICU patients [13]. An increase in plasma proinflammatory cytokine levels predicts mortality in patients with AKI [14]. Potential tubular and vascular factors, as well as inflammatory processes, are involved in the pathogenesis of AKI [15]. Inflammation is now believed to play a major role in the pathophysiology of AKI [16, 17]. The vascular endothelium plays a central role in the recruitment and migration of circulating inflammatory cells into sites of inflammation [18]. In addition, several authors have demonstrated the renoprotective effects of various anti-inflammatory therapies, for example, mycophenolate, alpha-melanocyte stimulating hormone (*α*-MSH) targeting the proinflammatory pathways that participate in the pathogenesis of AKI [19]. The present review focuses on inflammatory mediators like cytokines, chemokines, neutrophils, lymphocytes, natural killer (NK) cells, and macrophages in AKI (Figure 1).

Experimental studies in AKI have utilized ischemia-reperfusion, sepsis-endotoxemia, and nephrotoxic models [20]. These models of AKI are associated with a strong inflammatory reaction. It is hypothesized that in ischemia, sepsis, and nephrotoxic models that the initial insult results in morphological and/or functional changes in vascular endothelial cells and/or in tubular epithelium [15, 21]. Then, leukocytes including neutrophils, macrophages, natural killer cells, and lymphocytes infiltrate into the injured kidneys. The injury induces the generation of inflammatory mediators like cytokines and chemokines by tubular and endothelial cells which contribute to the recruiting of leukocytes into the kidneys (Table 1). Thus, inflammation plays an important role in the initiation and extension phases of AKI. This review will focus on the mediators of inflammation contributing to the pathogenesis of AKI.

## 2. Renal Endothelial Cells

Renal vascular endothelial cells initiate early inflammatory responses in the injured kidney because of direct contact with injurious agents [22]. Using intravital two-photon microscopy and the FVB-TIE2/GFP mouse [23], in which the endothelium is fluorescent, it has been demonstrated that ischemic injury damages the barrier function of endothelium resulting in disorganization of endothelial integrity including the partial disappearance of cell-cell borders and interruption of cell-cell contacts. Thus, endothelial disintegration increases vascular permeability and facilitates leukocyte infiltration into the renal parenchyma. Recent studies have investigated the leukocyte-endothelium interactions. Netrin-1 is an anti-inflammatory molecule first discovered in the brain. Netrin-1 plays an important role in the control of neuronal navigation during development of the nervous system and has also been shown to contribute to the patterning of developing epithelial tissues [24]. It has been determined that downregulation of netrin-1 in renal vascular endothelial cells in peritubular capillaries may promote endothelial cell activation resulting in infiltration of leukocytes into the kidney and tubular injury [25]. Sphingosine 1-phospate maintains endothelial cell integrity and inhibits lymphocyte extravasations via the sphingosine 1-phospate receptor. A recent study showed that a sphingosine 1-phospate-selective agonist ameliorates ischemic acute renal failure [26]. Additionally, renal endothelial prostacyclin synthesis may contribute to renal protection against endotoxemia-related AKI by maintaining the systemic and renal hemodynamic parameters [27]. Also, endothelial cells have a major role in the accumulation of leukocytes by expressing the adhesion molecules and chemokines (discussed in later sections).

## 3. Renal Tubular Epithelium

Renal tubular epithelium is a major site of cell injury and death during AKI. Several studies have suggested that renal tubular epithelial cells play a proinflammatory role in AKI. Interferon regulatory factor-1 (IRF-1) is a transcription factor known to activate proinflammatory genes, including interferons and chemokines. A recent study has demonstrated that IRF-1 is an early critical proinflammatory signal stimulated by reactive oxygen species during ischemic injury in vitro and in vivo and is produced within S3 proximal tubule cells. Transgenic knockout of IRF-1 ameliorated the impairment of renal function, morphologic injury, and inflammation after acute ischemia [28]. The Rho kinase pathway plays an important role in dedifferentiation of epithelial cells and infiltration of inflammatory cells. A study has investigated the blockade of the Rho kinase cascade within renal tubular cells and the interstitium. Inhibition of Rho kinase with the renally targeted Y27632-lysozyme conjugate strongly inhibits tubular damage, inflammation, and fibrogenesis induced by ischemia/reperfusion injury indicated by reduced staining of the dedifferentiation marker kidney injury marker-1 (KIM-1) and increased E-cadherin relative to controls [29]. KIM-1 is an immunoglobulin superfamily cell surface molecule expressed on epithelial cells after injury. Increased expression of KIM-1 on injured epithelial cells transforms the epithelial cells into phagocytes that can clear apoptotic and necrotic cells [30]. Phagocytosis of dead cells mediated by KIM-1 upregulation may influence the inflammatory response in AKI [30]. We will now review the roles of other mediators of inflammation such as complement, chemokines, cytokines, and toll-like receptors (TLRs) that regulate or are released by the renal tubular epithelial cells, renal endothelial cells, and inflammatory cells.

## 4. Cytokines

Many cytokines are released by leukocytes and renal tubular cells into the injured kidney and are important components of both the initiation and extension of inflammation in AKI. The proinflammatory cytokines/chemokines IFN*γ*, IL-2, IL-10, GM-CSF, TGF-*β*, CXCL1, IL-6, MIP-2, and MCP-1 are increased in the kidney in ischemic AKI [31–35]. 

Caspase-1 is a proinflammatory caspase via its activation of the cytokines IL-1*β* and IL-18. Administration of IL-1 receptor antagonist to mice is not protective against ischemic AKI [36]. IL-1*β* deficient mice are not protected against ischemic AKI [36]. Thus IL-18 was investigated as a potential mediator of the protection against ischemic AKI in caspase-1 deficient mice. IL-18 is a proinflammatory cytokine produced by proximal tubules [37], lymphocytes [38], neutrophils [39], and macrophages [40] in ischemic AKI. Activation of IL-18 by caspase-1 results in production of several cytokines and chemokines, activation of T helper cells, and proliferation of lymphocytes. It was demonstrated that caspase-1 deficient mice are functionally and histologically protected against ischemic AKI and that this protection is associated with decreased conversion of IL-18 precursor to the mature form in the kidney [41]. In this study, the administration of IL-18-neutralizing antiserum also protected against ischemic acute renal failure [41]. In subsequent studies, it was determined that IL-18 binding protein (a natural inhibitor of IL-18) overproducing transgenic mice are protected against ischemic AKI [42]. A recent study confirmed the pathogenic role of IL-18 in ischemic AKI in mice: pretreatment of wild-type mice with IL-18-binding protein is renoprotective and IL-18 deficient mice are protected against ischemic AKI [43]. IL-18-mediated ischemic AKI has neutrophil- and macrophage-independent mechanisms [39, 40]. 

Cisplatin-induced AKI is associated with increases in the cytokines IL-1*β*, IL-1*α*, IL-6, and IL-18 and neutrophil infiltration in the kidney [44]. However, inhibition of IL-1*β*, IL-6, and IL-18 and neutrophils by IL-1 receptor antagonist, IL-6 (−/−) mice, IL-18 antiserum, IL-18 binding protein transgenic mice, or neutrophil-depleting antibody respectively was not sufficient to prevent cisplatin-induced AKI [44]. 

Caspase-1 deficient mice are protected against cisplatin-induced AKI [45] and endotoxemia-induced AKI [46]. The mechanism of protection against cisplatin-induced AKI and endotoxemia-induced AKI in caspase-1 deficient mice remains to be determined. TNF-alpha is a potent proinflammatory cytokine and important mediator of inflammatory tissue damage. Cisplatin-induced AKI causes TNF-alpha synthesis. Cisplatin [47, 48] and endotoxin-induced [49] AKI is mediated in-part by TNF-alpha and the inhibition of the release or action of TNF-alpha protects the kidney from nephrotoxicity. 

Alpha-MSH is an important anti-inflammatory molecule that inhibits IL-8 and intracellular adhesion molecule-1 (ICAM-1) and significantly reduces renal injury after ischemia reperfusion in both wild-type and neutrophil depleted models [50, 51]. Recently, the administration of AP214 (an analogue of alpha-MSH) was reported to ameliorate sepsis-induced AKI and mortality in mice [52].

Interleukin-10 (IL-10) is a potent anti-inflammatory cytokine that inhibits inflammatory and cytotoxic pathways implicated in acute renal injury. IL-10 protects against ischemic AKI and cisplatin-induced AKI. IL-10 may act, in part, by inhibiting the maladaptive activation of genes that cause leukocyte activation and adhesion [53].

IL-6 is a proinflammatory cytokine that has been shown to mediate ischemic AKI [32, 54] and lung injury in mice with AKI [55]. IL-6 can stimulate target cells via a soluble form of the IL-6 Receptor in a process called trans-signaling [54]. The mechanism of the effect of IL-6 in ischemic AKI may be related to trans-signaling and STAT3 activation in renal tubular cells.

## 5. Chemokines

Chemokines are a large subgroup of cytokine-like molecules that play a major role in the recruitment of leukocytes in inflammation and in the regulation of T helper-1/T helper-2 immune responses. Chemokines have been divided into four subfamilies-CXC, CC, C, and CX_3_C-according to the number and spacing of conserved cysteine residues in their sequences [56]. Chemokines are induced by cytokines (TNF-*α* and IL-1*β*), complement activation, reactive oxygen species, NF-KB system, and TLR-related pathways [56]. It has been reported that various chemokines and chemokine receptors contribute to tissue injury in animal models of inflammatory kidney disease [56]. 

The chemokine receptor CCR1 regulates trafficking of macrophages and neutrophils to the kidney in a mouse model of renal ischemia-reperfusion injury [57]. Wild-type mice pretreated with the specific CCR1 antagonist BX471 and CCR1 deficient mice had fewer neutrophils and macrophages than control mice. Also, CCR1 deficient mice had reduced content of the CCR1 ligands CCL3 (MIP-1alpha) and CCL5 (RANTES). 

Proinflammatory cytokines increase expression of the CX_3_C chemokine, fractalkine, on injured endothelial cells. The fractalkine receptor (CX_3_CR1) is expressed on natural killer (NK) cells, monocytes, and some CD8+ T cells [18]. Fractalkine has a mucin-like stalk that extends the chemokine domain away from the endothelial cell surface enabling presentation of the CX_3_C-chemokine domain to leukocytes. Expression of fractalkine enables bypassing of the first 2 steps of the adhesion cascade (i.e., rolling and triggering) and mediates cell adhesion between circulating leukocytes and endothelial cells as well as extravasation of these cells. Thus, fractalkine serves the dual function of an adhesion molecule and a chemoattractant [18]. Fractalkine is a major chemoattractant for NK cells and monocytes but not neutrophils [58]. Fractalkine expression is increased in patients with renal tubulointerstitial inflammation with the strongest expression localized to vascular sites near to macrophage inflammation [59]. Fractalkine is a strong candidate for directing mononuclear cell infiltration induced by vascular injury [59]. Fractalkine expression is increased in the endothelium of large blood vessels, capillaries, and glomeruli in ischemic AKI and fractalkine receptor inhibition is protective against ischemic AKI [60]. Fractalkine expression is also increased in the blood vessels in mouse kidneys exposed to cisplatin [61]. However fractalkine inhibition did not protect against the functional and histological abnormalities in cisplatin-induced AKI in mice [61].

CXCL1 (also known as KC or IL-8), a prototypic CXC chemokine, is a neutrophil chemoattractant and attracts both neutrophils and T lymphocytes to the site of inflammation [62]. It has been previously demonstrated that CXCL1 is increased in the kidney in ischemic AKI [33, 34, 63]. Studies suggest that CXCL1 is a mediator of ischemic AKI. Injection of a neutralizing antibody to CXCL1 in mice results in decreased neutrophil infiltration in the kidney and protection against ischemic AKI [64]. CXCL1 binds to the chemokine receptors CXCR1 and CXCR2. In a rat model of renal transplantation, repertaxin, a CXCR2 inhibitor, prevents granulocyte infiltration in the kidney and prevents deterioration of kidney function [65]. CXCL1 is made by monocytes/macrophages, fibroblasts, keratinocytes, and endothelial cells [62, 66]. In ischemic AKI, CXCL1 staining was detected in macrophages and tubular epithelial cells. 

Macrophage depletion is known to be protective against ischemic AKI in mice [67, 68]. A key question is the mechanism of this protective effect. A possible mechanism of this protective effect is inhibition of production of CXCL1 by macrophages in ischemic AKI. Thus, it was determined whether macrophages are the source of CXCL1 in the kidney in ischemic AKI [42]. Inhibition of macrophage infiltration in the kidney using liposomal encapsulated clodronate prevented the increase in CXCL1 in the kidney suggesting that macrophages are the main source of CXCL1 in ischemic AKI. In view of the inhibition of CXCL1 production in the kidney by macrophage depletion and the documented protective effect of CXCL1 inhibition in ischemic AKI [64, 65], it is possible that part of the protective effect of macrophage depletion in ischemic AKI is mediated by inhibition of CXCL1. 

## 6. Adhesion Molecules

Adhesion molecules are required for leukocyte adhesion during inflammation. Leukocyte adhesion to endothelial cells leads to inflammation and extension of cellular injury [69]. ICAM-1 plays an important role in the pathophysiology of AKI [70, 71]. The administration of a monoclonal ICAM-1 antibody and ICAM-1 deficient mice is protected against renal ischemia [72, 73]. Pretreatment with topical polyethylene glycol hydrogel delivery of ICAM-1 antisense oligonucleotides demonstrated decreased ICAM-1 mRNA expression, reduced ICAM-1 protein staining, and decreased cellular damage in a murine partial nephrectomy/ischemia model [74]. 

The selectins and their ligands are other important adhesion molecules that participate in the inflammatory response. P-selectin is expressed as part of the inflammatory stimulus in platelets and endothelial cells, L-selectin is expressed in leukocytes and lymphocytes, and E-selectin is expressed in endothelium. Renal ischemia has been shown to be associated with upregulation of endothelial P-selectin but not L-selectin, with enhanced adhesion of neutrophils [75]. Studies have demonstrated that inhibition of P-selectin ameliorates the inflammatory response and ischemic AKI in rats and mice [76–78] and endotoxemia-induced AKI in rabbits [79]. A selectin ligand inhibitor, TBC-1269, was reported to provide protection against both short- and long-term effects of AKI in a pig model [78, 80]. E-selectin and its ligands are essential for extravasation of leukocytes in inflammation. CD147 (also known as Basigin or Bsg) is a membrane glycoprotein that belongs to the immunoglobulin superfamily and is a ligand for E-selectin. A recent study has indicated that CD147 is responsible for neutrophil recruitment in renal ischemia/reperfusion [81]. Compared with wild-type mice, CD147-deficient mice demonstrated significant suppression of neutrophil infiltration in the kidney after renal ischemia/reperfusion [81].

## 7. Complement System and Toll-Like Receptors

The complement system effectively identifies and clears hazardous agents as well as injured host cells. Uncontrolled complement activation can also contribute to tissue injury, however, and inhibition of this system may ameliorate many types of inflammatory injury. Complement activation in the kidney after ischemia-reperfusion was reported to occur primarily via the alternative pathway [82]. Selective inhibition of the alternative pathway provided protection from ischemic kidney injury [83]. Treatment with an inhibitory monoclonal antibody to mouse factor B, a necessary component of the alternative pathway, prevented activation of complement in the kidney after ischemia-reperfusion and protected the mice from necrotic and apoptotic tubular injury [84]. The complement inhibitor, complement receptor 1-related protein y (Crry), is expressed by proximal renal tubules on the basolateral membrane. Loss of polarity of Crry in the tubular epithelium preceded the activation of alternative pathway along the basolateral aspect of the tubular cells. After ischemia/reperfusion, altered renal tubular expression of Crry was reported to permit complement activation. Crry-deficient mice are more susceptible to ischemia, confirming the protective role of Crry expression [85]. A recent study showed that C3a (the first component of the alternative pathway) is required for the production of macrophage inflammatory factor (MIP-2/CXCL2) and keratinocyte-derived chemokine (KC/CXCL1/IL-8) by proximal tubular epithelial cells after renal ischemia/reperfusion. Selective antagonism of the C3a receptor significantly attenuated production of MIP-2 and KC, whereas C5a receptor antagonism and prevention of membrane attack complex (MAC) formation did not have a significant effect on MIP-2 and KC production [34]. 

TLRs are a family of transmembrane receptors that are widely expressed on leukocytes and kidney epithelial cells and regulate innate and adaptive immune responses. Renal tubular epithelial cells are known to express TLR-2 and 4, and expression of TLR-2 and TLR-4 was reported to increase during endotoxemic, ischemic, and nephrotoxic AKI [86]. TLR-2 deficient mice and mice treated with TLR-2 antisense oligonucleotides are protected from ischemic renal injury [87]. It has been demonstrated that systemic endotoxin has direct access to renal sites where TLR-4 is expressed [88]. A significant increase in TLR-4 expression by tubular epithelial cells and infiltrating leukocytes was demonstrated in the kidney following ischemia [88, 89]. TLR-4 (−/−) mice engrafted with wild-type hematopoietic cells had significantly lower serum creatinine and less tubular damage than wild-type mice reconstituted with TLR-4 (−/−) bone marrow [88]. Tamm-Horsfall protein (THP) is a glycoprotein with unclear functions expressed in thick ascending limbs of the kidney. Using THP knockout mice, the roles of THP and TLR-4 were investigated in renal ischemia-reperfusion injury. It was demonstrated that THP protects the kidney from ischemic injury by decreasing inflammation and altering TLR-4 expression during renal ischemia [90]. A recent study has investigated the effect of chloroquine, an inhibitor of TLR-3, 7, 8, and 9, in a polymicrobial sepsis mouse model [91]. Chloroquine administration attenuated the decline in renal function and serum pro- and anti-inflammatory cytokines like TNF-alpha and IL-10. Administration of an oligodeoxynucleotide inhibitor (H154) of TLR9 and TLR9-deficient mice demonstrated attenuation of AKI and a decrease in serum pro- and anti-inflammatory cytokines. Thus chloroquine and inhibition of TRP-9 protect from sepsis-induced AKI [91]. Another recent study demonstrated that TLR-4 signaling mediates inflammation and tissue injury in cisplatin-induced AKI [92]. Cisplatin-treated wild-type mice had significantly more renal dysfunction, histological damage, leukocyte infiltration in the kidney, and higher levels of cytokines in serum, kidney, and urine than cisplatin- treated mice with a targeted deletion of TLR-4.

## 8. Inflammatory Cells

### 8.1. Neutrophils

The adherence of neutrophils to the vascular endothelium is the first step in the extravasation of these cells into injured tissue. After adherence and chemotaxis, infiltrating neutrophils can release reactive oxygen species that damage the tubular cells [93].

The precise role of neutrophils in AKI has been addressed in many studies and remains controversial [94, 95]. Ischemic, nephrotoxic, and endotoxemia-induced AKI are associated with an increase in infiltrating neutrophils in the kidney [39, 41, 44, 89, 96]. There is evidence that neutrophils mediate tubular injury in AKI and play a key role in the development of acute renal failure [94]. This evidence is derived from studies that show an accumulation of neutrophils in ischemic AKI and studies demonstrating a beneficial role of anti-ICAM-1 therapy in AKI [73]. In addition, mice depleted of peripheral neutrophils by antineutrophil serum were protected against ischemic AKI [73]. Also, a recent study demonstrated that rapid CD44 upregulation on renal capillary endothelial cells mediates neutrophil recruitment to the postischemic tissue in a renal ischemia-perfusion mouse model. CD44 deficiency or administration of anti-CD44 to mice reduced the influx of neutrophils into the postischemic tissue, associated with renal function preservation [97]. 

However, in another study, rats depleted of peripheral neutrophils by antineutrophil serum were not protected against ischemic AKI [98]. Also, mice injected with the rat IgG2b monoclonal antibody RB6-8C5 that results in peripheral blood neutrophil depletion had a small (18%) reduction in serum creatinine during ischemic AKI but no reduction in the ATN score despite a lack of neutrophil infiltration in the kidney [39, 99]. In addition caspase-1 activity and IL-18 were still significantly increased in the kidney in neutrophil-depleted mice with AKI [39]. 

A recent study investigated the role of cathepsin G release by the activated neutrophils in renal ischemia-perfusion injury, using cathepsin G knockout mice. The study demonstrated that cathepsin G is required for sustained neutrophil-mediated inflammation and tissue injury after reperfusion of ischemic kidneys [100]. 

In summary, there are studies demonstrating both an injurious and noninjurious role of neutrophils in AKI. Interestingly, AKI is still seen in neutropenic patients suggesting that neutrophil depletion alone is not sufficient to protect against AKI.

### 8.2. Lymphocytes

The role of lymphocytes in AKI is an ongoing area of study. Lymphocytes have been shown to be important modulators of innate and adaptive inflammatory responses in AKI models [101]. In one study, mice with a combined deficiency of both CD4 and CD8 T cells were protected against ischemic AKI [102]. In a follow-up report by the same investigators, *nu/nu* mice deficient in both CD4 and CD8 T cells were also protected from AKI and adoptive transfer of T cells into these mice reconstituted renal injury [103]. CD4 knockout mice, but not CD8 knockout mice, were markedly protected against ischemic AKI and adoptive transfer of CD4 T cells into these mice restored renal injury [103, 104]. CD4 T cells can functionally differentiate into either a T helper 1 (IFN-gamma producing) or the counterbalancing T helper 2 (IL-4 producing) phenotype. A study of CD4 T cell subsets in ischemia reperfusion induced-AKI demonstrated that the T helper 1 phenotype is pathogenic and the T helper 2 phenotype can be protective [105]. Mice deficient in B lymphocytes alone are also protected against ischemic AKI [106]. The effect of T cells on vascular permeability in ischemic AKI in CD3, CD4, and CD8 T cell deficient mice was investigated and demonstrated that T cells directly contribute to the increased renal vascular permeability after ischemia-reperfusion, likely via T cell cytokine production (TNF-alpha and INF-gamma) [107]. In contrast, in another study, depletion of CD4 cells using the GK 1.5 antibody was not sufficient to prevent ischemic AKI in mice [38].

 The role for T lymphocytes in cisplatin-induced AKI was examined with T cell deficient (*nu/nu*) mice and CD4- and CD8-deficient mice and their wild-type littermates. T cell-deficient and CD4-deficient mice were protected from cisplatin-induced AKI and adoptive transfer of T cells into *nu/nu* mice enhanced renal dysfunction and tubular injury [108]. 

Recent studies have focused on the role of regulatory T cells (Tregs) in ischemic AKI. Tregs are a subtype of lymphocytes that have strong immunosuppressive and anti-inflammatory properties. Tregs are identified by their expression of CD4 and CD25 on the cell surface and upregulation of the FoxP3, a unique marker for Treg cells that is essential for their development and function [109]. Tregs have been reported to mediate ischemic AKI through IL-10 mediated suppression of the innate immune system [110]. Partial depletion of Tregs with an anti-CD25 monoclonal antibody worsened ischemic kidney damage and resulted in more neutrophil and macrophage infiltration in the kidney and increased innate cytokine transcription in the kidney. FoxP3+ Treg-deficient mice accumulated a greater number of leukocytes while Treg repletion significantly attenuated renal injury and leukocyte accumulation. Adoptive transfer of wild-type Tregs into RAG-1 knockout mice was sufficient to prevent kidney injury. However, transfer of IL-10 deficient Tregs did not prevent ischemic injury [110]. Pharmacological recruitment of Tregs is a potential future therapy for AKI.

### 8.3. Natural Killer Cells

NK cells are a type of lymphocyte that mediates innate immunity against pathogens and tumors via their ability to secrete cytokines [111]. NK cells are unique in their constitutive expression of receptors for cytokines and chemokines. NK cells in mice express mostly the same receptors as humans including NK 1.1. A model of NK cell activation in injured tissues has been proposed [112]. In this model, it is hypothesized that NK cells are recruited to sites of injury from the bloodstream. Once in the tissue, NK cells become activated and release cytokines like IL-18 [112]. In support of this hypothesis, it is known that NK cells play a role in numerous disease processes [113]. 

NK cells are increased in the kidney in adriamycin nephropathy but depletion of NK cells using antiasialo GM-1 antibody is not protective against adriamycin nephropathy [114]. A recent study investigated the role of NK cells in renal tubular epithelial cells in culture and in mice. The study demonstrated for the first time that NK cells can directly kill epithelial cells and that NK cells induce apoptosis in tubular epithelial cells and contribute to renal ischemia-reperfusion injury. NK cell depletion in wild-type mice was protective against AKI, while adoptive transfer of NK cells worsened AKI in NK cell, T cell, and B cell-null Rag-2 (−/−) gamma(c) (−/−) mice with AKI [115]. 

Natural killer T cells (NKT cells) are another interesting subtype of lymphocytes and have some of the characteristics of T and NK cells. NKT cells have regulatory functions by releasing cytokines and IFN-gamma. NKT cell activation was reported to mediate neutrophil and IFN-gamma production in ischemic AKI in mice [116]. It has been demonstrated that isoflurane anesthesia protected against ischemic AKI by reducing inflammation and modulating renal neutrophil, macrophage, and NKT cell influx in mice [117].

### 8.4. Macrophages

Macrophages are well-known sources of proinflammatory caspases and cytokines like IL-18 [118, 119] and IL-1*α* [120]. Infiltrating macrophages in the kidney have been described in various kidney diseases including glomerulonephritis, diabetic nephropathy, renal ablation and unilateral ureteric obstruction. In various models of glomerulonephritis, production of proinflammatory cytokines and chemokines, for example, iNOS, TNF-*α*, IL-1*β* MCP-1, MIF, and NK-kB by macrophages is thought to play a role in the pathogenesis of the disease [121]. Macrophage-mediated inflammation in the interstitium of the outer stripe of the outer medulla has been described early in the course of ischemic AKI in the rat [122, 123] and mouse [67, 68, 124]. Depletion of macrophages in the kidney during AKI using liposomal encapsulated clodronate [60, 67, 68] or genetic techniques [124] results in protection against ischemic AKI. An anti-B7-1 antibody blocked mononuclear cell adherence in vasa recta in rats and attenuated ischemic AKI both functionally and histologically [123]. Reduced postischemic macrophage infiltration and interstitial fibrosis are seen in osteopontin knockout mice [125]. 

The mechanism of protection against ischemic AKI by macrophage depletion is not known. To answer the question whether IL-18 from macrophages is contributing to the development of AKI, the effect of adoptive transfer of wild type and IL-18 deficient macrophages in macrophage-depleted mice with AKI was determined. The aim was to demonstrate that adoptive transfer of wild type macrophages reversed the protection produced by macrophage depletion and that adoptive transfer of macrophages deficient in IL-18 did not reverse the protection. Adoptive transfer of the peritoneal macrophages deficient in IL-18 reversed the protection against ischemic AKI to the same degree as wild type macrophages suggesting that ischemic AKI is independent of IL-18 production by macrophages [40].

Cisplatin-induced AKI is associated with increased renal myeloperoxidase, which is produced by neutrophils and macrophages [45, 108]. In vitro studies show that cisplatin administration increases macrophage-mediated cytotoxicity against neoplastic cells [126]. In addition, macrophage renal infiltration was recently shown to occur late in the course of cisplatin-induced AKI [127]. However, macrophage depletion using liposomal encappsulated clodronate was not protective in a mouse model of cisplatin-induced AKI [61].

### 8.5. Dendritic Cells

The principal DC function is the induction of adaptive immune responses, in particular those executed by T cells. Precursors of DCs are derived from the bone marrow and enter the kidney from the bloodstream. In the absence of “danger” signals like tissue damage or local inflammation, DCs will remain immature and fail to upregulate costimulatory molecules. Local inflammation or tissue damage can induce DC maturation, which results in the loss of phagocytic activity, expression of costimulatory molecules, and interaction with T cells. It is unknown whether these characteristics apply to DCs in the kidney [128].

DCs are abundantly present in the interstitium in normal mouse kidneys [129]. These DCs expressed the DC marker CD11c. Renal DCs resemble splenic DCs, but not peritoneal macrophages, in morphology, lysosomal content, phagocytic activity, microbicidal effector functions, expression of T cell costimulatory molecules, and ability to activate T cells. Renal DCs also express MHC class II and costimulatory molecules [129]. DCs form a contiguous network throughout the entire kidney [130].

Renal DCs have been reported to play an important role in ischemic AKI. Following kidney ischemia-perfusion, resident dentritic cells release TNF, IL-6, MCP-1, and RANTES and the depletion of DCs before ischemic injury significantly reduces TNF levels in the kidney [131]. 

There are interactions between NK cells and DCs that are important in the inflammatory response. Activated NK cells induce DC maturation. Immature DCs are vulnerable to autologous NK-cell-mediated cytolysis while mature DCs are protected. NK-cell-mediated DC activation is dependent on tumor necrosis factor-alpha (TNF-alpha) and interferon-gamma (IFN-gamma). In vitro, IL-12, IL-18, IL-15, and IFN-alpha/beta production by activated DCs augments NK-cell IFN-gamma production, proliferation, and cytotoxicity. [132].

## 9. Endoplasmic Reticulum Stress in the Kidney

The ER functions in the regulation of protein biosynthesis, protein folding, and the transport of synthesized proteins. The ER detects the presence of unfolded or misfolded proteins and protein chaperones catalyze protein folding [133]. The accumulation of unfolded and misfolded proteins in the ER lumen induces ER stress. ER stress results in an adaptive response known as the unfolded protein response (UPR) [134]. The UPR facilitates the adaptation to stress and attempts to reestablish normal ER function by activating calcium-dependent molecular chaperones like glucose-regulated protein-78 (GRP78) (also known as BiP) [134]. The production of reactive oxygen species (ROS) during oxidative stress results in protein misfolding in the ER Pharmacological activation of the UPR results in functional and histological protection against AKI in a mouse model [135, 136]. Bax inhibitor-1 (BI-1) is an ER protein that suppresses cell death. BI-1 knockout mice have worse ischemic liver and kidney injury compared to wild-type mice suggesting that the cytoprotective gene BI-1 provides intrinsic protection from ER stress [137]. The oxygen-regulated protein (ORP150) is an inducible ER chaperone. Renal tubular epithelial cells transfected with ORP150 were protected against hypoxic injury whereas inhibition of ORP150 made cells more susceptible to hypoxic injury [138]. Transcriptional regulators of genes involved in the inflammatory response, for example, nuclear factor-kB (NF-kB) are activated by ER stress [134]. In summary, ER stress in the kidney is a novel mediator of kidney injury.

## 10. Summary

In summary, in AKI, both renal endothelial cells and proximal tubular epithelial cells produce cytokines and chemokines that result in infiltration of inflammatory cells (neutrophils, lymphocytes, macrophages, NK cells, Treg cells) into the interstitium of the kidney (Figure 1). Inflammatory cells in the kidney produce pro- and anti-inflammatory cytokines that may further increase or decrease inflammation in the kidney. The mechanism by which adherent and infiltrating leukocytes cause ischemic tubular epithelial and endothelial injury is unclear, but likely involves the release of oxygen radicals [139, 140], the release of potent vasoconstrictors including the prostaglandins, leukotrienes, and thromboxanes [141], as well as direct endothelial injury via release of endothelin and a decrease in NO [142, 143].

## Figures and Tables

**Table 1:** Inflammatory mediators of AKI.

*Caspases*
Caspase-1

*Alpha-MSH*

*Cytokines*
(IFN-*γ*)
(TGF-*β*)
(TNF-*α*)
(IL-6)
(IL-10)
(IL-18)

*Chemokines*
(CXCL1 (also known as KC or IL-8 in mice))
(CX3CL1 (also known as fractalkine))
(CCR1 (Chemokine receptor-1) ligands MIP-1alpha and RANTES)
(MCP-1)
(MIP-2)

*Adhesion molecules*
(ICAM-1)
(P-selectin, E-selectin)
(CD147)

*Complement*
Alternative pathway
C3A

*TLRs*
TLR-2
TLR-4
TLR-9

*Neutrophils*

*Lymphocytes*
CD4
CD8
B cells
Tregs
NK cells

*Macrophages*

*Dendritic cells*
